# Staff Perspectives on Lessons Learned From an Interdisciplinary Memory Care Coordination Program

**DOI:** 10.1093/geroni/igab046.1263

**Published:** 2021-12-17

**Authors:** Inga Antonsdottir, Quincy Samus, Melissa Reuland, Deirdre Johnston, Morgan Spliedt, Danetta Sloan

**Affiliations:** 1 Johns Hopkins School of Nursing, Baltimore, Maryland, United States; 2 Johns Hopkins University School of Medicine, Baltimore, Maryland, United States; 3 Johns Hopkins University, Baltimore, Maryland, United States; 4 The Johns Hopkins University, Baltimore, Maryland, United States; 5 Johns Hopkins Bloomberg School of Public Health, Baltimore, Maryland, United States

## Abstract

MIND at Home is a home-based care coordination program for persons living with dementia (PLWD) and their informal care partners (CP). Assessments, care planning and coordination is delivered by trained non-clinical Memory Care Coordinators (MCCs), working together on an interdisciplinary team with nurses and geriatric psychiatrists. We report qualitative results from program staff (two nurses and eight MCCs) who implemented the program in the context of two clinical trials. Care team respondents answered open-ended questions covering 5 domains pertaining to: helpful skillsets; positive and challenging factors aspects of care coordination; barriers to care coordination for clients; and improvements suggestions/resources to strengthen the program. Compassion, finding common ground, listening, organization, and time management were reported as critical skills. Staff enjoyed team collaboration, being in and learning about the community, increasing CP confidence and mastery when caring for a PLWD. Reported challenges included documentation in EHR, accessing/navigating resources, driving long distances, unsafe neighborhoods, ambiguous assessment tools, and working with low engagement clients. Common barriers faced by clients (as reported by staff) were financial struggles/poverty, and lack of insurance coverage for needed services. Staff suggested several improvements: better communication strategies, integration with LTSS services and medical providers, 24-hour program hotline, continuous education for staff, simplified data collection and care delivery tracking process. This presentation on the experience of MIND at Home trained nurses and MCCs provides deep insight on how this and similar care coordination programs might be successfully implemented or strengthened.

